# 2-Meth­oxy­carbonyl-6-nitro­benzoic acid

**DOI:** 10.1107/S1600536812030462

**Published:** 2012-07-10

**Authors:** Zai-Sheng Lu, Guong-Zhou Zhu, Han Lu, Xiang-Shan Wang

**Affiliations:** a School of Chemistry and Engineering, Jiangsu Key Laboratory of Green Synthetic Chemistry for Functional Materials, Xuzhou Normal University, Xuzhou, Jiangsu 221116, People’s Republic of China

## Abstract

In the title compound, C_9_H_7_NO_6_, the dihedral angles between the benzene ring and its three substituents are 29.99 (8)° for the nitro, 67.09 (8)° for the carb­oxy and 32.48 (10)° for the meth­oxy­carbonyl group. In the crystal, one classical O—H⋯O and two nonclassical C—H⋯O contacts link adjacent mol­ecules, forming a three-dimensional structure.

## Related literature

For the bioactivity of the title compound, see: Xu & He (2010 ▶). For related structures, see: Glidewell *et al.* (2003 ▶); Wang *et al.* (2006 ▶).
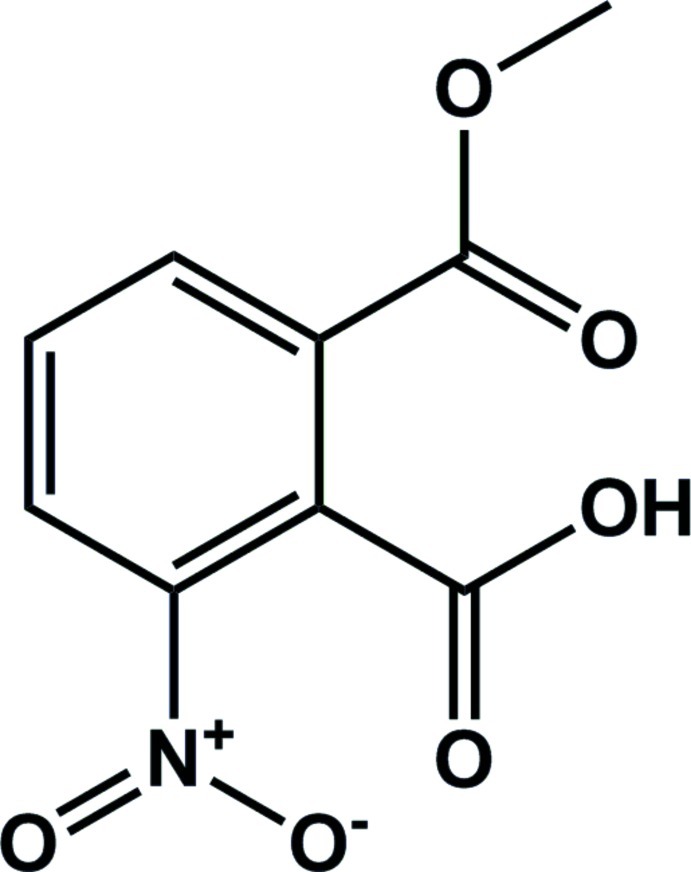


## Experimental

### 

#### Crystal data

C_9_H_7_NO_6_
*M* *_r_* = 225.16
Orthorhombic, 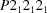

*a* = 7.647 (3) Å
*b* = 8.145 (3) Å
*c* = 15.583 (6) Å
*V* = 970.6 (7) Å^3^
*Z* = 4
Mo *K*α radiation
μ = 0.13 mm^−1^
*T* = 296 K
0.27 × 0.22 × 0.16 mm

#### Data collection

Bruker SMART CCD area-detector diffractometer
6820 measured reflections
1010 independent reflections
982 reflections with *I* > 2σ(*I*)
*R*_int_ = 0.021

#### Refinement

*R*[*F* ^2^ > 2σ(*F* ^2^)] = 0.027
*wR*(*F* ^2^) = 0.077
*S* = 1.05
1010 reflections
151 parameters
1 restraint
H atoms treated by a mixture of independent and constrained refinement
Δρ_max_ = 0.13 e Å^−3^
Δρ_min_ = −0.13 e Å^−3^

### 

Data collection: *SMART* (Bruker, 2001 ▶); cell refinement: *SAINT* (Bruker, 2001 ▶); data reduction: *SAINT*; program(s) used to solve structure: *SHELXS97* (Sheldrick, 2008 ▶); program(s) used to refine structure: *SHELXL97* (Sheldrick, 2008 ▶); molecular graphics: *SHELXTL* (Sheldrick, 2008 ▶); software used to prepare material for publication: *SHELXTL*.

## Supplementary Material

Crystal structure: contains datablock(s) global, I. DOI: 10.1107/S1600536812030462/sj5245sup1.cif

Structure factors: contains datablock(s) I. DOI: 10.1107/S1600536812030462/sj5245Isup2.hkl

Supplementary material file. DOI: 10.1107/S1600536812030462/sj5245Isup3.cml

Additional supplementary materials:  crystallographic information; 3D view; checkCIF report

## Figures and Tables

**Table 1:** Hydrogen-bond geometry (Å, °)

*D*—H⋯*A*	*D*—H	H⋯*A*	*D*⋯*A*	*D*—H⋯*A*
O4—H1⋯O5^i^	0.86 (1)	1.85 (1)	2.706 (2)	178 (3)
C9—H9*C*⋯O2^ii^	0.96	2.52	3.465 (3)	170
C9—H9*B*⋯O3^iii^	0.96	2.56	3.291 (3)	133

Symmetry codes: (i) 
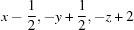
; (ii) 
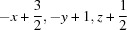
; (iii) 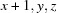.

**Fig. 1.**
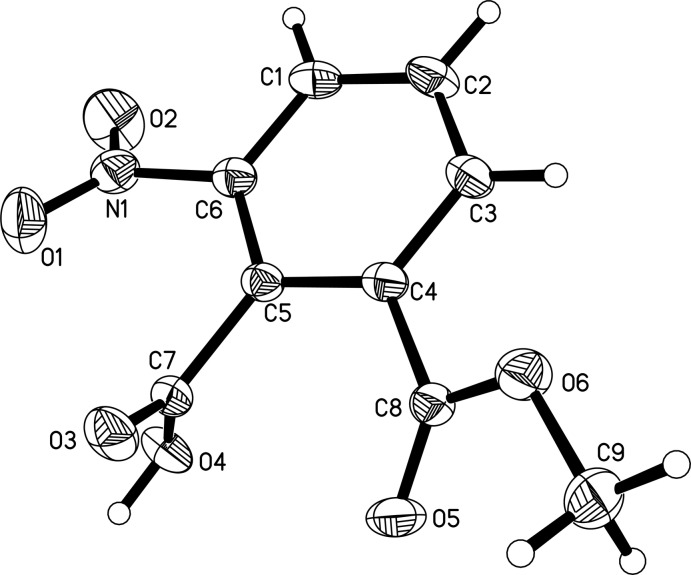
The structure of (I), showing 30% probability displacement ellipsoids and the atom-numbering scheme.

**Fig. 2.**
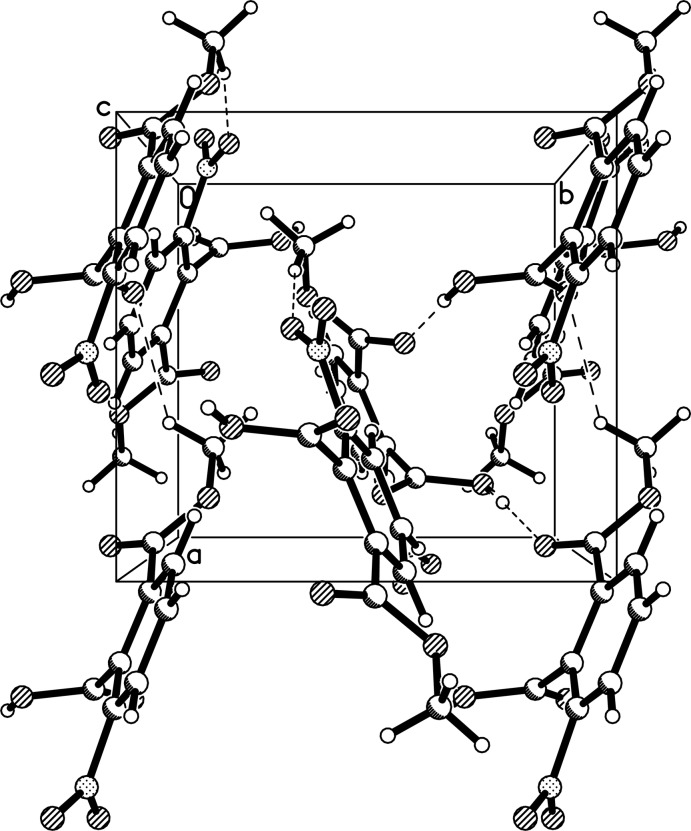
The crystal packing for (I).

## supplementary crystallographic information

### Comment

2-(Methoxycarbonyl)-6-nitrobenzoic acid is an important precursor to farm chemicals (Xu & He, 2010). The X-ray structures of 3-nitrophthalic acid (Glidewell *et al.*, 2003) and its organic adduct (Wang *et al.*, 2006) have been determined previously, however, to our knowledge, no structure of the title compound (I) has been reported. In the molecule of (I), Fig. 1, none of three substituents are coplanar with the benzene ring. The dihedral angles between benzene ring and these substituents are 29.99 (8)° for nitro group (N1/O1/O2), 67.09 (8)° for carboxylic acid (C7/O3/O4), and 32.48 (10)° for methoxycarbonyl (C8/O5/O6/C9), substituent respectively. This variation is likely to result from attempts to minimise steric hindrance between adjacent substituents. In the crystal structure, there are three hydrogen bonds, Table 1, one classical O4—H1···O5 and two nonclassical C9—H9B···O3 and C9—H9C···O2 contacts. These link adjacent molecules forming a three dimensional structure, Fig. 2.

### Experimental

A solution of 3-nitrophthalic acid (10.0 g) in acetic anhydride (15 ml) was refluxed for 1 h to obtain 3-nitrophthalic anhydride (8.0 g). Then the product was dissolved in 50 ml anhydrous methanol and stirred at room temperature for 2 h, after which 1 ml concentrated sulfuric acid was dropped into the mixture, refluxed for 24 h, cooled and filtered. The resulting solid was dimethyl 3-nitrophthalate. The filtrate was concentrated and then chromatographed over silica gel (mobile phase: n-hexane:acetone = 1:3). The title compound (I) was collected from mobile phase (1.0 g, m.p. 429–431 K). Crystals of (I) suitable for X-ray diffraction were obtained by slow evaporation of a toluene solution.

### Refinement

The H atom bonded to O4 was located in a difference Fourier map and refined freely. All other H atoms were placed in calculated positions, with C—H = 0.93–0.98 Å and included in the final cycles of refinement using a riding model, with U_iso_(H) = 1.2U_eq_(parent atom). In the absence of significant anomalous dispersion effects, Friedel pairs were averaged.

### Crystal data

**Table:**

C_9_H_7_NO_6_	*D*_x_ = 1.541 Mg m^−^^3^
*M**_r_* = 225.16	Melting point = 429–431 K
Orthorhombic, *P*2_1_2_1_2_1_	Mo *K*α radiation, λ = 0.71073 Å
Hall symbol: P 2ac 2ab	Cell parameters from 6294 reflections
*a* = 7.647 (3) Å	θ = 2.6–27.1°
*b* = 8.145 (3) Å	µ = 0.13 mm^−^^1^
*c* = 15.583 (6) Å	*T* = 296 K
*V* = 970.6 (7) Å^3^	Block, colourless
*Z* = 4	0.27 × 0.22 × 0.16 mm
*F*(000) = 464	

### Data collection

**Table:**

Bruker SMART CCD area-detector diffractometer	982 reflections with *I* > 2σ(*I*)
Radiation source: fine-focus sealed tube	*R*_int_ = 0.021
Graphite monochromator	θ_max_ = 25.0°, θ_min_ = 3.0°
φ and ω scans	*h* = −9→9
6820 measured reflections	*k* = −8→9
1010 independent reflections	*l* = −18→18

### Refinement

**Table:**

Refinement on *F*^2^	Secondary atom site location: difference Fourier map
Least-squares matrix: full	Hydrogen site location: inferred from neighbouring sites
*R*[*F*^2^ > 2σ(*F*^2^)] = 0.027	H atoms treated by a mixture of independent and constrained refinement
*wR*(*F*^2^) = 0.077	*w* = 1/[σ^2^(*F*_o_^2^) + (0.0524*P*)^2^ + 0.1444*P*] where *P* = (*F*_o_^2^ + 2*F*_c_^2^)/3
*S* = 1.05	(Δ/σ)_max_ = 0.001
1010 reflections	Δρ_max_ = 0.13 e Å^−^^3^
151 parameters	Δρ_min_ = −0.13 e Å^−^^3^
1 restraint	Extinction correction: *SHELXL97* (Sheldrick, 2008), Fc^*^=kFc[1+0.001xFc^2^λ^3^/sin(2θ)]^-1/4^
Primary atom site location: structure-invariant direct methods	Extinction coefficient: 0.058 (7)

### Special details

**Table:**

Geometry. All e.s.d.'s (except the e.s.d. in the dihedral angle between two l.s. planes) are estimated using the full covariance matrix. The cell e.s.d.'s are taken into account individually in the estimation of e.s.d.'s in distances, angles and torsion angles; correlations between e.s.d.'s in cell parameters are only used when they are defined by crystal symmetry. An approximate (isotropic) treatment of cell e.s.d.'s is used for estimating e.s.d.'s involving l.s. planes.
Refinement. Refinement of *F*^2^ against ALL reflections. The weighted *R*-factor *wR* and goodness of fit *S* are based on *F*^2^, conventional *R*-factors *R* are based on *F*, with *F* set to zero for negative *F*^2^. The threshold expression of *F*^2^ > σ(*F*^2^) is used only for calculating *R*-factors(gt) *etc*. and is not relevant to the choice of reflections for refinement. *R*-factors based on *F*^2^ are statistically about twice as large as those based on *F*, and *R*-factors based on ALL data will be even larger.

### Fractional atomic coordinates and isotropic or equivalent isotropic displacement parameters (Å^2^)

**Table:**

	*x*	*y*	*z*	*U*_iso_*/*U*_eq_	
O6	1.15166 (19)	0.64183 (18)	0.90968 (8)	0.0467 (4)	
O5	1.0302 (2)	0.41107 (19)	0.95864 (9)	0.0504 (4)	
O4	0.6725 (2)	0.22147 (17)	0.89827 (8)	0.0481 (4)	
O3	0.6525 (2)	0.4616 (2)	0.96756 (9)	0.0503 (4)	
C5	0.7676 (2)	0.4546 (2)	0.82508 (10)	0.0319 (4)	
O2	0.4446 (3)	0.3360 (3)	0.67750 (11)	0.0790 (6)	
C2	0.9167 (3)	0.5774 (3)	0.67363 (12)	0.0481 (5)	
H2A	0.9677	0.6167	0.6235	0.058*	
O1	0.4073 (2)	0.4102 (3)	0.80807 (11)	0.0694 (5)	
C6	0.6778 (2)	0.4551 (2)	0.74720 (11)	0.0359 (4)	
N1	0.4967 (3)	0.3961 (2)	0.74398 (12)	0.0466 (4)	
C8	1.0429 (2)	0.5175 (2)	0.90536 (11)	0.0343 (4)	
C7	0.6900 (2)	0.3825 (2)	0.90552 (11)	0.0353 (4)	
C4	0.9356 (2)	0.5210 (2)	0.82510 (10)	0.0342 (4)	
C9	1.2706 (3)	0.6459 (3)	0.98203 (13)	0.0532 (6)	
H9A	1.3540	0.7327	0.9741	0.064*	
H9B	1.3310	0.5429	0.9861	0.064*	
H9C	1.2057	0.6649	1.0338	0.064*	
C3	1.0080 (3)	0.5825 (3)	0.74992 (13)	0.0430 (5)	
H3A	1.1197	0.6277	0.7510	0.052*	
C1	0.7503 (3)	0.5143 (2)	0.67198 (11)	0.0436 (5)	
H1A	0.6872	0.5112	0.6210	0.052*	
H1	0.630 (4)	0.178 (3)	0.9437 (12)	0.078 (9)*	

### Atomic displacement parameters (Å^2^)

**Table:**

	*U*^11^	*U*^22^	*U*^33^	*U*^12^	*U*^13^	*U*^23^
O6	0.0477 (8)	0.0497 (8)	0.0428 (7)	−0.0100 (7)	−0.0081 (6)	0.0050 (6)
O5	0.0555 (8)	0.0542 (8)	0.0414 (7)	−0.0066 (7)	−0.0148 (7)	0.0176 (7)
O4	0.0640 (9)	0.0431 (7)	0.0372 (7)	−0.0043 (7)	0.0154 (7)	0.0068 (6)
O3	0.0594 (9)	0.0603 (9)	0.0311 (7)	0.0006 (8)	0.0088 (6)	−0.0060 (6)
C5	0.0369 (8)	0.0321 (8)	0.0267 (8)	0.0043 (7)	0.0019 (7)	0.0008 (7)
O2	0.0743 (11)	0.1103 (15)	0.0524 (9)	−0.0313 (12)	−0.0165 (9)	−0.0122 (10)
C2	0.0598 (12)	0.0531 (12)	0.0314 (9)	−0.0055 (10)	0.0045 (9)	0.0121 (8)
O1	0.0423 (8)	0.1073 (15)	0.0587 (9)	−0.0063 (10)	0.0043 (8)	−0.0097 (10)
C6	0.0399 (9)	0.0363 (9)	0.0315 (8)	0.0017 (8)	−0.0011 (8)	0.0015 (7)
N1	0.0461 (8)	0.0542 (10)	0.0396 (8)	−0.0007 (8)	−0.0091 (7)	0.0012 (8)
C8	0.0350 (8)	0.0374 (9)	0.0304 (8)	0.0036 (8)	0.0021 (7)	0.0026 (8)
C7	0.0341 (8)	0.0443 (10)	0.0275 (8)	0.0014 (8)	0.0016 (8)	0.0021 (8)
C4	0.0397 (9)	0.0339 (8)	0.0290 (8)	0.0022 (8)	−0.0002 (7)	0.0042 (7)
C9	0.0470 (11)	0.0652 (13)	0.0473 (10)	−0.0074 (11)	−0.0104 (9)	−0.0036 (10)
C3	0.0447 (9)	0.0479 (11)	0.0364 (8)	−0.0054 (9)	0.0029 (7)	0.0111 (8)
C1	0.0568 (11)	0.0468 (10)	0.0271 (8)	0.0025 (9)	−0.0064 (8)	0.0055 (8)

### Geometric parameters (Å, º)

**Table:**

O6—C8	1.312 (2)	C2—H2A	0.9300
O6—C9	1.449 (2)	O1—N1	1.216 (3)
O5—C8	1.204 (2)	C6—C1	1.383 (3)
O4—C7	1.323 (3)	C6—N1	1.467 (3)
O4—H1	0.856 (10)	C8—C4	1.496 (2)
O3—C7	1.197 (2)	C4—C3	1.389 (3)
C5—C4	1.394 (3)	C9—H9A	0.9600
C5—C6	1.394 (2)	C9—H9B	0.9600
C5—C7	1.506 (2)	C9—H9C	0.9600
O2—N1	1.213 (2)	C3—H3A	0.9300
C2—C1	1.373 (3)	C1—H1A	0.9300
C2—C3	1.380 (3)		
			
C8—O6—C9	117.11 (15)	O3—C7—C5	123.81 (18)
C7—O4—H1	112 (2)	O4—C7—C5	110.80 (15)
C4—C5—C6	116.91 (16)	C3—C4—C5	120.48 (17)
C4—C5—C7	120.96 (15)	C3—C4—C8	119.57 (17)
C6—C5—C7	122.11 (16)	C5—C4—C8	119.86 (15)
C1—C2—C3	119.78 (18)	O6—C9—H9A	109.5
C1—C2—H2A	120.1	O6—C9—H9B	109.5
C3—C2—H2A	120.1	H9A—C9—H9B	109.5
C1—C6—C5	122.78 (18)	O6—C9—H9C	109.5
C1—C6—N1	117.60 (17)	H9A—C9—H9C	109.5
C5—C6—N1	119.58 (16)	H9B—C9—H9C	109.5
O2—N1—O1	123.7 (2)	C2—C3—C4	120.93 (19)
O2—N1—C6	118.15 (19)	C2—C3—H3A	119.5
O1—N1—C6	118.16 (17)	C4—C3—H3A	119.5
O5—C8—O6	124.84 (17)	C2—C1—C6	119.08 (18)
O5—C8—C4	123.11 (17)	C2—C1—H1A	120.5
O6—C8—C4	112.05 (14)	C6—C1—H1A	120.5
O3—C7—O4	125.37 (18)		
			
C4—C5—C6—C1	1.6 (3)	C6—C5—C4—C3	−0.7 (3)
C7—C5—C6—C1	−177.04 (16)	C7—C5—C4—C3	177.95 (18)
C4—C5—C6—N1	−176.33 (16)	C6—C5—C4—C8	−177.14 (16)
C7—C5—C6—N1	5.0 (3)	C7—C5—C4—C8	1.5 (2)
C1—C6—N1—O2	30.9 (3)	O5—C8—C4—C3	−145.9 (2)
C5—C6—N1—O2	−151.0 (2)	O6—C8—C4—C3	33.4 (2)
C1—C6—N1—O1	−149.0 (2)	O5—C8—C4—C5	30.5 (3)
C5—C6—N1—O1	29.0 (3)	O6—C8—C4—C5	−150.10 (16)
C9—O6—C8—O5	3.2 (3)	C1—C2—C3—C4	1.5 (3)
C9—O6—C8—C4	−176.16 (16)	C5—C4—C3—C2	−0.8 (3)
C4—C5—C7—O3	67.3 (2)	C8—C4—C3—C2	175.65 (18)
C6—C5—C7—O3	−114.1 (2)	C3—C2—C1—C6	−0.6 (3)
C4—C5—C7—O4	−111.59 (18)	C5—C6—C1—C2	−0.9 (3)
C6—C5—C7—O4	67.0 (2)	N1—C6—C1—C2	177.02 (18)

### Hydrogen-bond geometry (Å, º)

**Table:**

*D*—H···*A*	*D*—H	H···*A*	*D*···*A*	*D*—H···*A*
O4—H1···O5^i^	0.86 (1)	1.85 (1)	2.706 (2)	178 (3)
C9—H9*C*···O2^ii^	0.96	2.52	3.465 (3)	170
C9—H9*B*···O3^iii^	0.96	2.56	3.291 (3)	133

Symmetry codes: (i) *x*−1/2, −*y*+1/2, −*z*+2; (ii) −*x*+3/2, −*y*+1, *z*+1/2; (iii) *x*+1, *y*, *z*.
